# Susceptibility to auditory feedback manipulations and individual variability

**DOI:** 10.1371/journal.pone.0323201

**Published:** 2025-05-07

**Authors:** Muge Ozker, Peter Hagoort

**Affiliations:** 1 Max Planck Institute for Psycholinguistics, Nijmegen, Netherlands; 2 Donders Institute for Brain, Cognition and Behaviour, Nijmegen, Netherlands.; National Taiwan Normal University, TAIWAN

## Abstract

Monitoring auditory feedback from hearing one’s own voice is important for fluent and precise speech production as it enables the detection and correction of speech errors. This influence is evident when auditory feedback is manipulated, such as through delayed auditory feedback (DAF), which affects speech fluency by slowing speech rate, or pitch-perturbed auditory feedback (PAF), which affects vocalization leading to changes in voice pitch. Previous studies have tested both DAF and PAF in clinical populations and showed that susceptibility to these manipulations varied largely across individuals with different disorders. However, it remains unclear whether this variability stems from different task manipulations, as no single study has systematically tested both types of feedback manipulations within the same population. DAF and PAF affect different aspects of speech likely engaging distinct neural mechanisms. It remains uncertain whether individuals highly susceptible to one type of manipulation will also be susceptible to the other. To address this, we examined neurotypical individuals instead of clinical populations, allowing us to better control background variability. In addition to task manipulations, we were also interested in why individuals within a population vary in their susceptibility under each task manipulation. One possible explanation for individual differences in susceptibility is that some individuals rely more on auditory feedback, making them more sensitive to disruptions, while others depend more on alternative sensory modalities, rendering them less affected. Visual feedback, such as seeing one’s own mouth movements, has been shown to improve speech production in clinical populations with impairments. We aimed to test whether providing visual feedback as an alternative sensory modality could similarly reduce the effects of altered auditory feedback on speech in neurotypical individuals, particularly among those who may rely more on non-auditory sensory feedback. We recorded voice samples from 40 neurotypical participants during DAF and PAF tasks. In the DAF task, participants repeated sentences while experiencing delayed feedback, which significantly reduced their speech rate. In the PAF task, participants phonated a sustained vowel sound and experienced unexpected pitch perturbations in their auditory feedback. In most trials, they adjusted their voice pitch in the opposite direction to compensate for the perturbation. We assessed susceptibility to DAF and PAF by examining speech rate and peak amplitude of the compensatory response, respectively. Participants highly susceptible to DAF experienced notable speech rate reductions, while those highly susceptible to PAF exhibited larger pitch adjustments. Susceptibility varied widely among participants, with no consistent overlap between those sensitive to DAF and those sensitive to PAF, supporting distinct processing mechanisms for these different manipulations. Additionally, to examine the effect of visual feedback on speech production, we focused on the DAF task, as it involves visible mouth movements during sentence repetition. In some trials, participants received visual feedback by watching themselves speak through a webcam. Contrary to expectations, this immediate visual feedback did not alleviate but rather strengthened the disruptive effects of DAF, further reducing speech rate.

## Introduction

Auditory feedback from hearing one’s own voice during speech production plays an important role in speech monitoring as it allows for the detection and instant correction of speech errors. Disruptions to auditory feedback can significantly impact speech output, as evidenced by how post lingually deafened individuals may develop fluency problems [1,2]. To study the influence of auditory feedback, researchers commonly manipulate its timing or acoustic properties. Delaying auditory feedback (DAF) during speech production is recognized to induce dysfluencies reminiscent of stuttering, causing a decrease in speech rate [3–5]. Likewise, perturbing the fundamental frequency of auditory feedback (PAF) leads to adjustments in the voice pitch, typically in the opposite direction of the perturbation [6–15].

Research with clinical populations has shown that susceptibility to these auditory feedback manipulations varies widely. For example, DAF has been found to cause more dysfluencies in individuals with schizophrenia [16] and autism spectrum disorder [17], compared to neurotypical controls, indicating heightened susceptibility to DAF in these clinical groups. In contrast, patients with conduction aphasia show reduced susceptibility to DAF [18]. While DAF typically induces stutter-like speech in neurotypical individuals [5], it has been found to improve fluency in individuals who stutter [19]. Regarding PAF, compensatory vocal responses are significantly reduced in post-stroke aphasia patients [20] but are amplified in patients with Parkinson’s disease [21] compared to healthy individuals. To better understand this wide range of susceptibility in clinical populations, it is crucial to first examine variability within healthy populations. Studies with neurotypical individuals have also documented variable susceptibility to both DAF [22–25] and PAF [26–28], yet the extent and causes of this variability remain unclear. First objective of our study was to systematically explore these individual differences in susceptibility to DAF and PAF in neurotypical individuals.

DAF and PAF affect speech in fundamentally different ways, each engaging distinct mechanisms within the speech production system. DAF introduces a temporal discrepancy between articulation and auditory feedback, disrupting coordination during sequenced speech production (timing, rate, and fluency). It often leads to slowing down of speech, resetting syllables, prolonging vowels, or pausing—actions that appear to be efforts to realign articulation with delayed auditory feedback. In contrast, PAF directly affects vocalization during sustained phonation, leading to voice pitch adjustments that aim to correct the mismatch between intended and perceived pitch. Together, DAF and PAF highlight different behaviors speakers exhibit to manage disruptions in auditory feedback. However, it remains uncertain whether susceptibility to one type of manipulation predicts susceptibility to the other. Some individuals may be selectively affected by DAF, while others by PAF, suggesting that susceptibility could either be specific to a particular type of manipulation or reflect a more general sensitivity to auditory feedback disruptions. Thus, our second objective was to examine whether susceptibility to DAF and PAF are correlated.

Another important question is why some individuals are highly susceptible to auditory feedback manipulations, while others can continue speaking or vocalizing with minimal influence, seemingly able to disregard these manipulations. One hypothesis is that individuals may have different sensory preferences, relying more heavily on certain sensory modalities over others [29]. During speech production, sensory feedback comes from multiple sources, including auditory feedback (hearing one’s own voice), proprioceptive feedback (from the muscles and joints involved in articulation), and somatosensory feedback (from oral structures like the lips and tongue). Those more affected by auditory feedback manipulations may rely predominantly on auditory feedback, while those less affected may rely more on other sensory feedback. An alternative source of sensory feedback is visual feedback (seeing one’s own mouth movements while speaking), which is not naturally available during speech production. However, evidence from clinical populations demonstrated that providing visual feedback by allowing participants to see their own mouth movements in a mirror can improve impaired speech production in stuttering and aphasia [30,31]. A previous DAF study in neurotypical individuals found that providing immediate visual feedback also reduced speech dysfluencies caused by DAF. In line with the sensory preference hypothesis, this improvement was most notable among speakers who were already less susceptible to DAF, suggesting that they may indeed rely less on auditory feedback and can utilize alternative sensory feedback sources to a greater extent [32]. Our third objective was to replicate this finding by providing immediate visual feedback alongside DAF to examine its impact on fluency and the extent to which it might mitigate DAF’s effects for individuals with varying susceptibility profiles. We focused on the DAF task for this purpose, as unlike sustained vowel phonation in the PAF task, the DAF task involves visible mouth movements during sentence repetition. However, it is important to note that viewing one’s own mouth movements is not the only form of visual feedback; different types of visual feedback can be used in a PAF task to examine its effect on vocalization. For example, in a previous study, participants viewed their real-time pitch contours on a screen during sustained phonation, and findings showed that this visual feedback helped stabilize their voice pitch [33].

In summary, we recorded voice samples from 40 participants in a gender balanced, neurotypical population during DAF and PAF tasks and investigated: 1) interindividual differences in susceptibility to auditory feedback manipulations, measured by speech rate for DAF and peak amplitude of the compensatory response for PAF, 2) the relationship between susceptibility profiles for DAF and PAF, and 3) the potential for immediate visual feedback —allowing participants to watch themselves speak through a webcam—to improve speech fluency under DAF conditions.

## Materials and methods

### Participants

40 native Dutch speakers (20 females, age: 24.5 ± 5) without a known history of hearing or language impairments were included in the study after providing written informed consent prior to the start of the experiments (Participant recruitment period: 13/04/2022 - 01/08/2022). The research was approved by Radboud University’s Faculty of Social Sciences (ethics application ECSW-2020–046) and complied with the Declaration of Helsinki.

### Delayed auditory feedback experiment

#### Stimuli and equipment.

Audio recordings of 10 sentences spoken by a female native Dutch speaker were presented to the participants. The sentences consisted of 10–12 words and 15–18 syllables, with an average duration of 4 seconds. Stimulus presentation was executed using Matlab Psychtoolbox-3 on an HP Intel Core i7 laptop running Windows. Participants repeated the sentences, and their voices were recorded and played back using a Focusrite Scarlett 2i2 audio interface and a Sennheiser DT 790 closed headset equipped with a dynamic microphone. The recorded audio was delayed using the Psychtoolbox function ‘PsychPortAudio’. To provide visual feedback, a webcam captured a video feed of the participants’ faces. On randomly selected trials, this video feed was displayed on the laptop screen, allowing participants to watch themselves in real time as they spoke. The activation and deactivation of the webcam was controlled using the Psychtoolbox function ‘Screen’. High-quality recordings (48000Hz) of participants’ voices were obtained using a Zoom H6 recorder and used for analysis.

#### Task.

**Fig 1:**
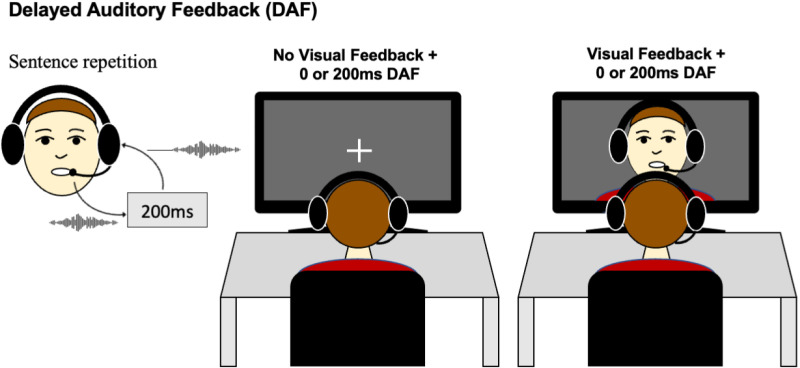
Illustration of the delayed auditory feedback (DAF) experiment.

Participants first listened passively to the auditorily presented sentences. 1 second after the sentence offset, they were prompted to repeat them aloud. During this repetition phase, auditory feedback was presented either simultaneously or with 200 ms delay (DAF) (Fig 1). In half of the trials, participants received immediate visual feedback (VF) synchronous with their articulation, in addition to auditory feedback. During these trials, the laptop’s webcam activated, and the participants watched themselves on the screen as they spoke. For trials with no visual feedback, participants fixated on a crosshair presented in the middle of the screen. For trials with visual feedback, participants were instructed to watch their own mouth movements on the screen. Thus, the experiment consisted of four different conditions: no DAF, DAF, no DAF + VF and DAF + VF. Each sentence was repeated twice for each condition, which resulted in a total of 80 trials. The trials for these four conditions were presented in a random order.

#### Analysis.

Participants’ audio recordings were analyzed in Praat. For each trial, sentence onset and offset were manually marked to calculate the sentence duration. Speech rate was calculated by dividing the sentence duration by the number of syllables contained in the sentence. Voice intensity and voice pitch contours were calculated using automated scripts.

#### LME model.

Linear mixed-effects models (lmer function in R) [34] were used to test the effects of two experimental manipulations (auditory feedback delay: 0 or 200 ms, visual feedback: present or absent) and two additional factors (gender and trial order) on three continuous outcome variables (speech rate, voice intensity, and voice pitch). Gender and trial order were added to the models because these factors were shown to influence responses in previous studies [35,36]. Voice intensity and pitch values were separately scaled within each gender group by subtracting the mean and dividing by the standard deviation before the LME analysis. Because different individuals may have different baseline speech rates, voice intensity and pitch, or may be affected differently by experimental manipulations, participants were added as a random effect using a random intercept and slope model. In addition, different sentence stimuli were also added as a random effect using a random intercept and slope model to account for any different effects that the 10 different sentence stimuli may have on the outcome variables. Models were estimated using REML, and the degrees of freedom (df), t-values, and p-values were calculated according to the Satterthwaite approximation. Before model estimation, we centered the categorical factors in our LME models around 0 to make the intercept represent the average effect across the conditions. For instance, the categorical factor *Delay* had two levels: absent and present. By centering, we coded the levels as -0.5 for absent and + 0.5 for present, which allowed the intercept to represent the overall mean effect across both conditions. This approach made the interpretation of the model results more intuitive and straightforward. To ensure model convergence, we scaled the ordinal factor *Trial order* (e.g., 1, 2, 3, …). This step was necessary because unscaled ordinal variables with large ranges can lead to numerical instability during optimization. Scaling *Trial order* brought all factors to a comparable scale, which improved numerical stability and facilitated convergence.

### Perturbed auditory feedback experiment

#### Stimuli and equipment.

Auditory feedback was perturbed during sustained phonation by shifting the voice fundamental frequency (F0: pitch) by either 100 or 200 cents in the upward or downward direction for 300 milliseconds, starting at a random latency (1400–1900 milliseconds) following the phonation onset. Stimulus presentation was executed using Matlab Psychtoolbox-3 on an HP Intel Core i7 laptop running Windows. Participants’ voices were recorded and played back using a Focusrite Scarlett 2i2 audio interface and a Sennheiser DT 790 closed headset equipped with a dynamic microphone. F0 perturbation was applied using the Matlab Audio Toolbox plugin object *PitchShifter*. Matlab simultaneously recorded the auditory feedback delivered through the headset speakers and the voice signal captured by the microphone, enabling precise temporal alignment of the two signals.

#### Task.

**Fig 2:**
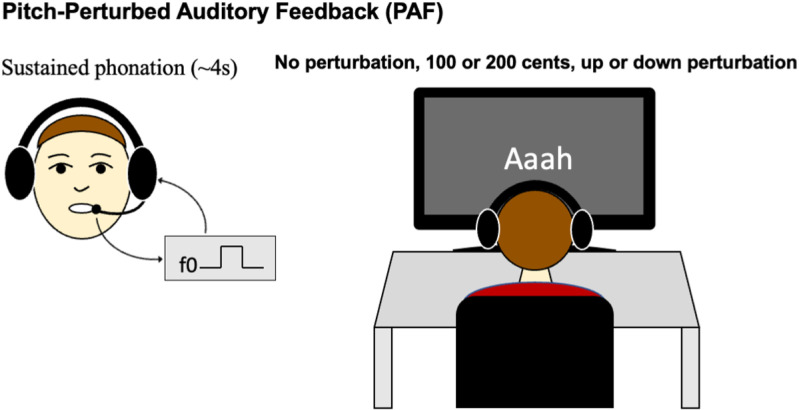
Illustration of the pitch-perturbed auditory feedback (PAF) experiment.

Participants phonated the vowel /a/ for 3.5–4 seconds. A text “Aaah” was presented on the screen to prompt the participants to phonate either with (+/-100 or + /-200 cents) or without perturbation (Fig 2). Thus, the experiment included five conditions: no PAF, + 100 PAF, + 200 PAF, -100 PAF, and -200 PAF. Each condition consisted of 30 trials, resulting in a total of 150 trials. The trials for these five conditions were presented in a random order.

#### Analysis.

Pitch of the participants’ voice was determined using the autocorrelation method in Praat [37]. Pitch contours for each trial were exported to Matlab for further analysis. Trials that contained large gaps or sharp discontinuities in their pitch track due to algorithm failure were excluded from the analysis. Overall, 5% of all trials were removed for this reason. Pitch contours were epoched from -200 to + 800 milliseconds with respect to the perturbation onset and were converted from Hertz to cents scale using the following formula: **F0(cents) = 1200 x log**_**2**_ **(F/ F**_**baseline**_)

Average pitch from -100–0 milliseconds before the perturbation onset was used as the baseline (F_baseline_). Each perturbation trial was first classified as having an opposing or a following response by performing linear regression on the pitch contour from +50 to + 350 milliseconds after perturbation. Response direction was determined by the slope of the fitted line, with positive slope indicating a following and negative slope indicating an opposing response for the upward perturbation condition and vice versa for the downward perturbation condition as in [38]. If the slope of the fitted line was not significant (p > 0.05), then the corresponding trial was classified as a *nonresponse*. After classification of trials as opposing and following, the peak amplitude for each trial was determined by identifying the maximum value of the pitch contour within the + 200 to + 500 milliseconds time window after perturbation.

#### LME model.

Linear mixed effects (lmer in R) models were used to test the effects of two experimental manipulations (perturbation direction: up or down, perturbation magnitude: 100 or 200 cents) and two additional factors (gender and trial order) on the peak amplitude of the compensatory response. Because different individuals may have different baseline voice pitch, or may be affected differently by experimental manipulations, participants were added as a random effect using a random intercept and slope model. To calculate each participant’s susceptibility to perturbed auditory feedback (*see* *Susceptibility Index Calculation*), we fitted an additional LME model. This model predicted the peak amplitude based on fixed effects for perturbation (present or absent, regardless of perturbation magnitude and direction), gender, and trial order, with participant as a random effect. Before model estimation, we centered the categorical factors in our LME models around 0 to make the intercept represent the overall mean effect across conditions. Additionally, we scaled the ordinal factor *Trial order*, which brought all factors to a comparable scale improving numerical stability and facilitating model convergence. Linear mixed models were estimated using REML, and the degrees of freedom (df), t-values, and p-values were calculated according to the Satterthwaite approximation.

### Susceptibility index calculation

Linear mixed effect (LME) models were fitted to speech rate and peak amplitude of the compensatory response for the delayed and perturbed auditory feedback experiments, respectively (*see LME Model*). In these models, participants were added as random factors using a random intercept and slope. LME models provide a fixed-effect slope that represents the average effect of the predictor variable (i.e., *auditory feedback delay’* for DAF or *perturbation’* for PAF experiments) across all participants. They also provide random slopes per each participant, which capture how much individual participants deviate from the population-level fixed-effect slope. The random slope values of the fixed effect *‘auditory feedback delay’* were used as individual susceptibility indices for the DAF experiment. Similarly, the random slope values of the fixed effect *‘perturbation’* were used as individual susceptibility indices for the PAF experiment.

## Results

### Delayed auditory feedback experiment

In the delayed auditory feedback experiment, participants repeated out loud auditorily presented sentences. Their voice recordings were analyzed to extract three response variables including speech rate, voice intensity and voice pitch.

**Fig 3:**
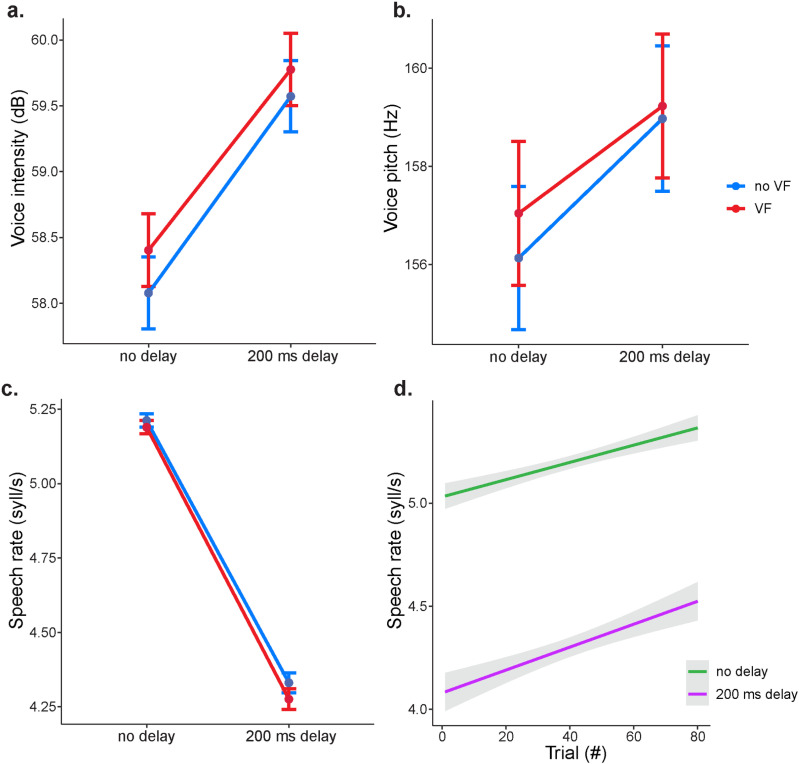
Effect of delayed auditory feedback on speech. a) Voice intensity, b) Voice pitch and c) Speech rate averaged across trials and participants for simultaneous (0ms) and delayed (200ms) auditory feedback when visual feedback is present (red) and absent (blue). Error bars indicate standard error of the mean across trials. d) The effect of trial order on speech rate for simultaneous (green) and 200ms delayed (purple) auditory feedback. Shaded regions indicate 95% confidence intervals.

Linear mixed effects models were fitted to these response variables to examine the effects of auditory feedback delay (no delay versus 200ms delay), visual feedback (webcam on versus off), gender and trial order. To account for overall differences across participants and 10 different sentence stimuli, these were added to the models as random factors (*see Methods*). There was a significant negative effect of delay on speech rate and a positive effect on voice intensity and pitch. These results suggest that hearing one’s own voice with 200ms delay decreases speech rate (no DAF: 5.2 ± 0.6 *vs.* DAF: 4.3 ± 0.9 syllables per second) and increases voice intensity (no DAF: 58.2 ± 7.8 *vs.* DAF: 59.7 ± 7.7 decibels) and voice pitch (no DAF: 156.6 ± 41.3 *vs.* DAF: 159.1 ± 41.6 Hertz) (Fig 3a-c, Tables 1–3). In contrary to our expectation, there was a significant positive effect of visual feedback on all response variables, suggesting that seeing one’s own face during speech production further decreases speech rate (no VF: 4.77 ± 0.9 *vs.* VF: 4.73 ± 0.9 syllables per second), and increases voice intensity (no VF: 58.8 ± 7.7 *vs.* VF: 59.1 ± 7.8 decibels) and voice pitch (no VF: 157.5 ± 41.5 *vs.* VF: 158.1 ± 41.4 Hertz), reinforcing the effects of DAF on speech. The interaction of delay and visual feedback was not significant, suggesting that visual feedback decreased speech rate, and increased voice intensity and pitch regardless of whether auditory feedback was delayed.

**Table 1:** Results of a linear mixed effects model for speech rate.

*Fixed Effects*	*Estimate*	*SE*	*df*	*t*	*p*
(Intercept)	4.75	0.11	46.15	43.06	<2e-16
**Delay**	**-0.89**	**0.1**	**40.7**	**-8.6**	**1.1x10** ^**-10**^
**VF**	**-0.04**	**0.01**	**39.06**	**-2.58**	**0.01**
Gender	-0.29	0.19	38	-1.5	0.14
**Trial order**	**0.12**	**0.02**	**34.87**	**6.5**	**1.67x10** ^**-7**^
Delay x VF	-0.02	0.02	3013	-0.93	0.35

The fixed effects were delay (0 vs 200 ms), visual feedback (present or absent), gender, trial order and interaction of delay and visual feedback. Participants and different sentence stimuli were included in the model as random factors. For each effect, the model estimates (in units of syllables per seconds) for that factor are shown relative to intercept. Significant effects are shown in **bold**.

**Table 2:** Results of a linear mixed effects model for voice intensity.

*Fixed Effects*	*Estimate*	*SE*	*df*	*t*	*p*
(Intercept)	0.0003	0.16	42.07	0.002	0.99
**Delay**	**0.22**	**0.03**	**40.78**	**6.32**	**1.52x10** ^**-7**^
**VF**	**0.04**	**0.01**	**39.02**	**3.48**	**0.001**
Gender	0.0001	0.3	38	0	0.99
Trial order	0.02	0.02	37.93	1.29	0.21
Delay x VF	-0.03	0.02	3011	-1.37	0.17

The fixed effects were delay (0 vs 200 ms), visual feedback (present or absent), gender, trial order and interaction of delay and visual feedback. Participants and different sentence stimuli were included in the model as random factors. For each effect, the model estimates (in units of decibels) for that factor are shown relative to intercept. Significant effects are shown in **bold**.

**Table 3:** Results of a linear mixed effects model for voice pitch.

*Fixed Effects*	*Estimate*	*SE*	*df*	*t*	*p*
(Intercept)	0.00007	0.16	43.25	0	0.99
**Delay**	**0.14**	**0.03**	**36.57**	**4.6**	**5x10** ^**-5**^
**VF**	**0.03**	**0.013**	**38.68**	**2.42**	**0.02**
Gender	-0.002	0.3	37.99	-0.005	0.99
Trial order	-0.027	0.02	37.85	-1.37	0.18
Delay x VF	-0.03	0.03	8.99	-1.08	0.31

The fixed effects were delay (0 vs 200 ms), visual feedback (present or absent), gender, trial order and interaction of delay and visual feedback. Participants and different sentence stimuli were included in the model as random factors. For each effect, the model estimates (in units of Hertz) for that factor are shown relative to intercept. Significant effects are shown in **bold**.

Since females naturally have a higher voice intensity (Male: 54.5 ± 7.5 *vs.* Female: 63.4 ± 4.9 decibels) and pitch (Male: 120.3 ± 15.9 *vs.* Female: 195.4 ± 19.3 Hertz) compared to males, intensity and pitch values were separately scaled within each gender group by subtracting the mean and dividing by the standard deviation before the LME analysis. Gender did not have a significant effect on any of the response variables. Trial order had a significant positive effect on speech rate but no effect on voice intensity and pitch, suggesting that participants spoke faster as the experiment progressed, but they maintained a stable voice intensity and pitch throughout the experiment (Fig 3d).

Increased voice intensity and pitch are commonly reported effects of DAF, which were previously attributed to heightened muscular tension as individuals seek to counteract the experimental interference [4]. Another possibility is that participants unconsciously increase vocal effort to compensate for the disruption introduced by DAF, resulting in heightened intensity and pitch. This response might reflect a general effort to maintain clarity in speech under challenging feedback conditions.

**Fig 4:**
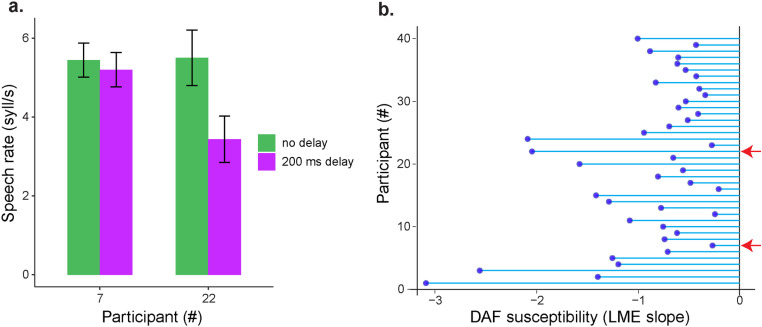
Individual variability in susceptibility to delayed auditory feedback. a) **Comparison of speech rates during simultaneous (green bars) and 200ms delayed (purple bars) auditory feedback in two different participants.** b) **Random slope values from the LME model which represent the effect of auditory feedback delay on speech rate for different participants. Red arrows indicate the participants that were shown in panel** a.

Additionally, during DAF, individuals often slow down or reset their articulation in an effort to realign their articulation with the delayed feedback, resulting in reduced speech rate [4,5]. As these latter actions are indicative of attempts to reduce the asynchrony between their articulation and the perceived auditory feedback, we focused on speech rate, rather than voice intensity and pitch, to evaluate the susceptibility profiles of different individuals to DAF. As an example, Fig 4a shows the speech rates from two participants during sentence production with simultaneous and delayed auditory feedback. While delayed feedback caused only a small decrease in speech rate in one participant (Participant #7; no DAF: 5.44 ± 0.4 *vs.* DAF: 5.2 ± 0.4 syllables per second), it caused a much larger decrease in another participant (Participant #22; no DAF: 5.5 ± 0.7 *vs.* DAF: 3.4 ± 0.6 syllables per second). Across participants, there was a considerable variability in susceptibility to delay, measured by the decrease in speech rate during 200ms DAF compared to no DAF condition (LME random slope values ranging from -0.2 to -3.1) (Fig 4b).

### Perturbed auditory feedback experiment

**Fig 5:**
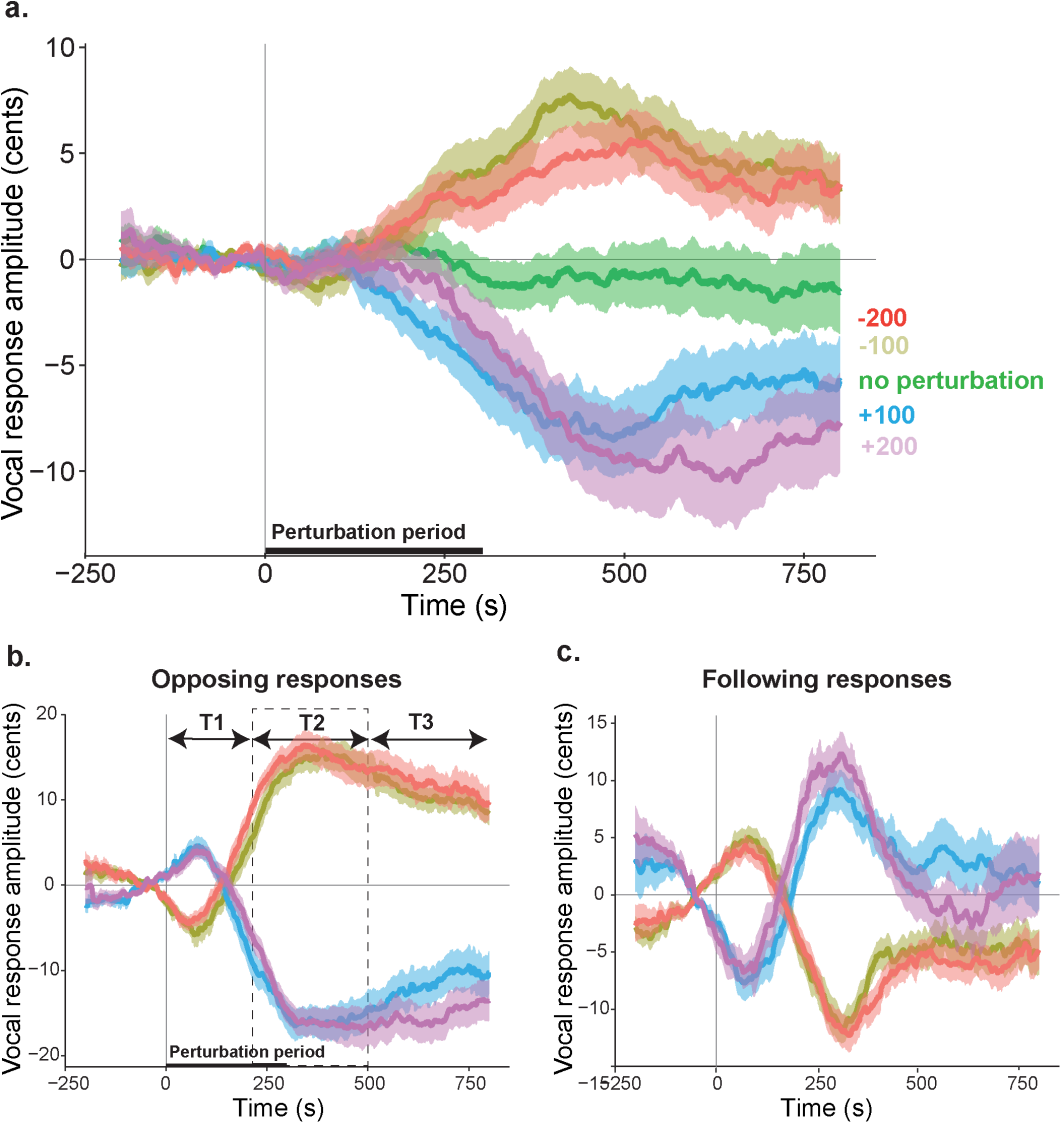
Vocal responses to F0 perturbation. a) Pitch contours during the phonation of the vowel /a/ with no perturbation, 100-cent and 200-cent perturbation in the upward and downward directions averaged across participants. Shaded regions indicate standard error of the mean across participants. b) Pitch contours of compensatory (opposing) vocal responses during the phonation of the vowel /a/ with 100-cent and 200-cent perturbation in the upward and downward directions averaged across participants. Shaded regions indicate standard error of the mean across participants. T1: 0 to 200, T2: 200 to 500 and T3: 500 to 800 milliseconds indicate different time periods with respect to the perturbation onset. Black dashed rectangle highlights time period T2 within which the maximum vocal responses are determined for the LME analysis c) Pitch contours of following vocal responses averaged across participants with shaded regions indicating standard error of the mean.

In the perturbed auditory feedback experiment, participants phonated the vowel /a/ for 3.5–4 seconds. During sustained phonation, the fundamental frequency (pitch) of their voice was perturbed 100 or 200 cents either in the upward or the downward direction for 300ms. Their voice recordings were analyzed to test how participants responded to perturbed auditory feedback. Examining the average pitch contours revealed that participants slightly shifted their voice pitch in the opposite direction of the perturbation (Fig 5a). These compensatory vocal responses reached a maximum at ~ 520 milliseconds following the perturbation onset, and they were very small compared to the perturbation magnitudes (Mean across participants: 7.7 cents (-100), 8.6 cents (+100), 5.6 cents (-200) and 10.5 cents (+200)).

Even though vocal responses were in the opposite direction of the perturbation when averaged across all trials, closely inspecting individual trials revealed that participants not always opposed but also followed the perturbation by shifting their voice pitch in the same direction of the perturbation. Categorizing each trial as opposing or following (*see Methods*) confirmed that while the majority of vocal responses were opposing, still a substantial percentage were following responses (Table 4).

**Table 4:** Percentage of different vocal response types for different F0 perturbation conditions.

Condition	Nonresponse	Opposing	Following
-200	8%	50%	42%
-100	7%	59%	34%
+100	11%	55%	34%
+200	10%	52%	38%

Since opposing responses reflect vocal compensation and error correction, the remainder of the analyses focused on opposing responses (Fig 5b), and not on following responses (Fig 5c). Inspecting the voice pitch contours for the opposing responses revealed different response phases after the perturbation onset. In the first phase, immediately after perturbation onset (**T1**: 0–200 ms), responses slightly increased in the same direction with the perturbation. However, in the second phase (**T2:** 200–500 ms), responses changed direction and increased in the opposite direction of the perturbation, with the peak amplitude occurring during this phase. In the last phase (**T3:** 500–800 ms), responses slightly decreased and remained sustained.

Vocal responses within the first 100–150 milliseconds after pitch perturbations are not considered as compensatory and error correction related [39]. This is because it takes a longer time for the auditory system to process the vocal output and inform the motor system to make the necessary corrections [40]. We therefore focused in the 200–500 millisecond period (**T2**) after the perturbation onset and determined the maximum value of the pitch contour within this period to assess the peak amplitude for each trial.

**Fig 6:**
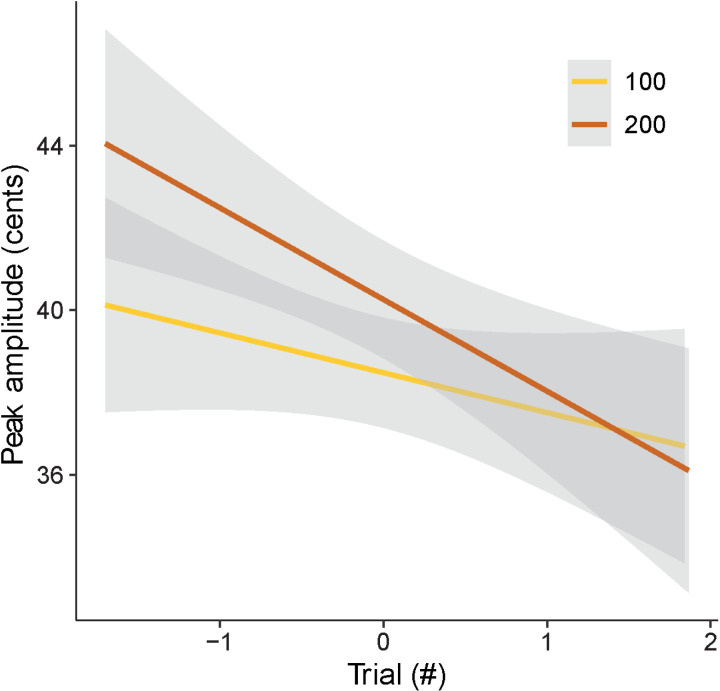
Effects of trial order on the peak amplitude of the compensatory response. Change in peak amplitude with the progression of trials for 100-cent (yellow) and 200-cent (brown) perturbation. Shaded regions indicate 95% confidence intervals.

An LME model was fitted to the peak amplitudes to examine the effects of perturbation direction (up or down), perturbation magnitude (100 or 200 cents), gender and trial order. To account for overall differences across participants, participant was added to the model as a random factor. None of the effects were significant (Table 5), except for a negative effect of trial order on the peak amplitude (p = 0.004), which suggests that participants made smaller compensatory vocal responses for the perturbation after repeated exposure to perturbed auditory feedback (Fig 6).

**Table 5:** Results of a linear mixed effects model for the peak amplitude of the compensatory response.

Fixed Effects	Estimate	Std. Error	df	t	p
(Intercept)	39.42	2.07	37.95	18.97	<2e-16
Perturbation magnitude	1.18	0.85	2392	1.38	0.16
Perturbation direction	0.63	1.12	28.16	0.56	0.58
Gender	-6.65	4.15	37.94	-1.6	0.12
**Trial order**	**-1.24**	**0.43**	**2402**	**-2.9**	**0.004**

The fixed effects were the perturbation magnitude (100 vs. 200 cents), perturbation direction (upward or downward), gender and trial order. Participants were included in the model as a random factor. For each effect, the model estimates (in units of cents) for that factor are shown relative to intercept.

Since perturbation direction (up or down) or perturbation magnitude (100 or 200 cents) did not have a significant effect on the peak amplitude, all perturbation trials were combined regardless of perturbation magnitude or direction. An LME model was fitted to the peak amplitudes to examine the effects of F0 perturbation (non-perturbed or perturbed), gender and trial order. Again, participants were added to the model as a random factor. There was a significant effect of perturbation on the peak amplitude (t = 3.64, p = 0.005; Table 6). There was also a marginal effect of gender suggesting that male participants made slightly larger compensatory responses compared to females (t = -2.03, p = 0.05). Additionally, there was a significant effect of trial order, once again showing that participants made smaller compensatory vocal responses for the perturbation after repeated exposure to perturbed auditory feedback (t = -2.26, p = 0.02).

**Table 6:** Results of a linear mixed effects model for the peak amplitude of the compensatory responses to all F0 perturbations.

Fixed Effects	Estimate	Std. Error	df	t	p
(Intercept)	37.64	1.95	37.99	19.27	<2e-16
**Perturbation**	**3.71**	**1.02**	**38.05**	**3.64**	**0.0008**
**Gender**	**-7.93**	**3.9**	**37.84**	**-2.03**	**0.05**
**Trial order**	**-0.76**	**0.34**	**3529**	**-2.26**	**0.02**

The fixed effects were the perturbation (present or absent), gender and trial order. Participants were included in the model as a random factor. For each effect, the model estimates (in units of cents) for that factor are shown relative to intercept. Significant effects are shown in **bold**.

**Fig 7:**
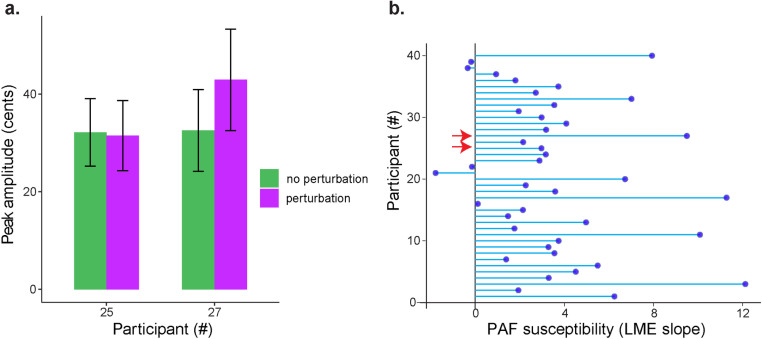
Individual variability in susceptibility to F0 perturbed auditory feedback. a) **Comparison of peak amplitudes during non-perturbed (green bars) and perturbed (purple bars) auditory feedback in two different participants.** b) Random **slope values from the LME model which represent the effect of perturbation on the peak amplitude for different participants. Red arrows indicate the participants that were shown in panel** a.

As an example, Fig 7a shows the peak amplitudes from two participants during phonation with perturbed and non-perturbed auditory feedback. While F0 perturbation did not affect peak amplitude in one participant (Participant #25; no PAF: 32.2 ± 13.8 *vs.* PAF: 31.5 ± 14.4 cents), it caused much larger increase in the other participant (Participant #27; no PAF: 32.5 ± 16.7 *vs.* PAF: 42.9 ± 20.7 cents).

To examine the interindividual variability in susceptibility to perturbed auditory feedback across participants, a susceptibility index was determined for each participant using the random slope value of the fixed effect ‘*perturbation’* from the fitted LME model. Across participants, there was a considerable variability in susceptibility to F0 perturbation, measured by an increase in peak amplitude during perturbation compared to no perturbation (LME random slope values ranging from -1.7 to 12.1) (Fig 7b).

### Comparison of susceptibilities to DAF and PAF

**Fig 8:**
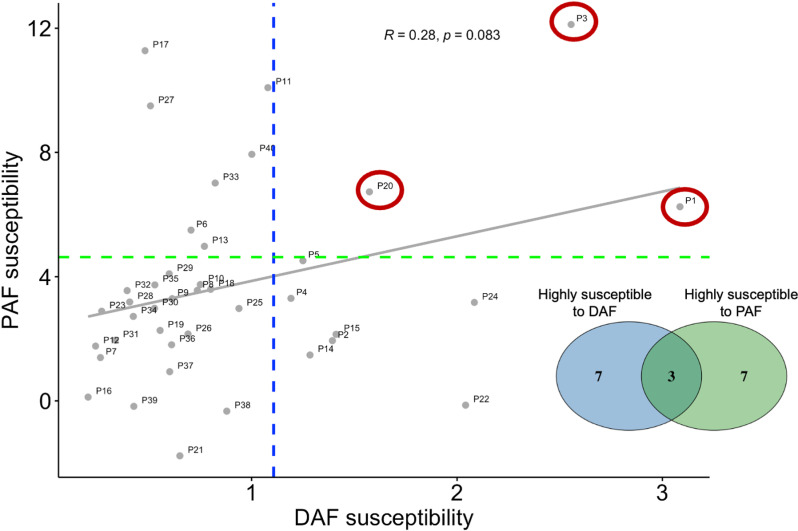
Correlation between DAF and PAF susceptibilities. Gray circles represent individual participants (P1–P40) and their susceptibility to DAF and PAF. The gray fitted line illustrates the lack of a significant correlation between susceptibilities in the two tasks. Dashed lines mark the 75th percentile thresholds, with participants above these thresholds classified as highly susceptible to DAF (blue) and PAF (green). The Venn diagram highlights that, of the 10 highly susceptible participants in each task, only three were highly susceptible to both DAF and PAF (circled in red).

Finally, to examine whether susceptibility to delayed and perturbed auditory feedback is correlated, we compared susceptibility indices (LME slopes in Figs 4b and 7b) across participants. In the DAF experiment, all slope values were negative, indicating that participants reduced their speech rate to varying degrees when subjected to a 200 ms delay (Fig 4b). In contrast, PAF slopes were mostly positive, reflecting an increase in voice pitch in response to pitch perturbation. However, a few participants had negative slopes, indicating a greater increase in voice pitch when no perturbation was present (Fig 7b). To standardize susceptibility measures, we used absolute LME slope values for the DAF experiment, ensuring that larger slope values consistently indicate greater susceptibility in both tasks. We found no significant correlation between susceptibility indices in the two tasks (Spearman correlation: r = 0.28, p = 0.08, Fig 8).

Next, we classified participants above the 75th percentile of susceptibility indices in each experiment as highly susceptible. This approach identified 10 out of 40 participants as *highly susceptible* in each experiment, but only 3 participants were classified as highly susceptible in both experiments. These results suggest that susceptibility to DAF and PAF may be independent, with individuals highly susceptible to one manipulation not necessarily being susceptible to the other.

## Discussion

This study highlights that individuals vary in their susceptibility to auditory feedback manipulations. Using both delayed auditory feedback (DAF) and pitch-perturbed auditory feedback (PAF) paradigms, we found susceptibility to DAF does not necessarily align with susceptibility to PAF, indicating separate processing mechanisms for each manipulation type.

### Different compensatory mechanisms for DAF and PAF

Theoretical models of speech production propose that during speech production, motor control regions issue a motor command to the articulators and concurrently transmit an efference copy of this command to the auditory control regions. The efference copy acts as a predictive signal that inhibits the sensory response to one’s own speech. If there is a mismatch between the prediction and the auditory feedback of the produced speech, e.g., when auditory feedback is perturbed, an error signal will be generated to adjust the motor command and correct the speech error [41–43]. In this study, we predicted that DAF and PAF elicit different types of disruptions in speech, which are possibly processed differently. Indeed, a previous meta-analysis study reviewed fMRI investigations that used frequency-altered, delayed, and masked auditory feedback, and found only a small activation overlap in the anterior auditory cortex [44]. This observation suggests that distinct neural systems are involved in detecting and compensating for the disruptions induced by these different manipulations. Our behavioral results provided further evidence supporting these neuroimaging findings by showing that susceptibility to DAF does not necessarily predict susceptibility to PAF, such that individuals may be strongly affected by one type of manipulation while remaining largely unaffected by the other.

We measured susceptibility to DAF by examining speech rate, as individuals more susceptible to DAF typically experience greater difficulty maintaining fluency, resulting in more pronounced speech slowing. For PAF, susceptibility was assessed based on the peak amplitude of the compensatory response: individuals more affected by PAF struggle to maintain stable phonation, showing larger deviations in their ongoing voice pitch. It is important to clarify the distinction between compensatory responses during DAF and PAF. In the case of PAF, speakers exhibit clear compensations by actively opposing the perturbation to minimize the mismatch between their intended and perceived voice pitch. These adjustments are quantifiable in both direction and magnitude, allowing precise measurement of how much the voice pitch changes in the opposite direction of the applied perturbation. In contrast, responses to DAF do not follow such a straightforward compensation pattern. We propose that the slowing of speech during DAF—manifested as syllable resetting, vowel prolongation, or pauses—is an attempt to address the temporal mismatch between articulation and auditory feedback. However, this type of response does not constitute compensation or reflect reactive feedback control in the same way as observed in PAF. If durational perturbations had been applied to specific speech segments (e.g., syllable onset, nucleus, or coda) by compressing or stretching them [45–49], we might have observed compensatory adjustments relative to the perturbation, such as lengthening or shortening of consonants or vowels. These compensatory responses, with measurable direction and magnitude, would have been more directly comparable to the compensatory responses seen in PAF. Furthermore, applying durational perturbations, as opposed to pitch perturbations, would have enabled a direct comparison of how temporal and acoustic properties of auditory feedback are processed. However, our ability to explore these effects was constrained by the nature of DAF, which was applied globally, shifting the timing of the entire utterance rather than altering temporal features of specific speech segments.

### Visual feedback does not improve fluency during DAF

Our results demonstrated a substantial variability in susceptibility to the effects of auditory feedback manipulations. However, it is not clear what determines the susceptibility of different individuals. Previous studies considered sensory modality preference, suggesting that individuals who rely more heavily on auditory feedback compared to somatosensory feedback during speech production are more susceptible to auditory feedback manipulations [50]. To test this idea, a previous study simultaneously altered auditory and somatosensory feedback by applying formant perturbation and mechanical loads to the jaw during speech production. Indeed, there was an inverse relationship between reliance on auditory versus somatosensory feedback, indicating that individuals who compensated more for one perturbation tended to compensate less for the other [29]. Stronger reliance on auditory feedback compared to somatosensory feedback might explain the greater susceptibility to altered auditory feedback. However, our experiments did not include somatosensory perturbations to test this hypothesis directly. Findings from formant perturbation studies may not apply to our DAF and PAF paradigms, as speakers may weigh auditory and somatosensory feedback differently for different perturbations. Formant perturbations primarily disrupt articulation, which may be more closely tied to somatosensory feedback. Also, while formant perturbations primarily target segmental speech features such as phonetic and phonological characteristics of individual sounds, DAF and PAF target suprasegmental speech features—temporal and pitch control, respectively—which operate over larger linguistic units such as syllables, words, or entire phrases. Further research is needed to determine how these suprasegmental speech control processes interact with somatosensory sensory feedback.

DAF introduces an asynchrony between the auditory and somatosensory feedback, and compensatory attempts to correct this asynchrony disrupt fluency, resulting in slower speech. In our experiment, we used DAF at 200 milliseconds latency, which is known to result in maximal disruption in healthy individuals, independent of speech rate or the length of utterance [51,52]. Instead of additionally manipulating somatosensory feedback, we tested whether an alternative form of sensory feedback that is synchronous with articulation (i.e., immediate visual feedback) can mitigate the disruptive effects of delayed auditory feedback. We expected that less susceptible individuals, if they indeed rely less on auditory feedback, would adapt strategies to overcome the effects of DAF by disregarding auditory feedback and focusing on visual feedback. However, in contrary to our expectations, immediate visual feedback intensified the disruptive effects of DAF. A prior investigation revealed that introducing delayed visual feedback alongside DAF also intensified the disruptive effects of DAF, suggesting that synchronizing visual feedback with delayed auditory feedback instead of articulation may also not provide a solution [53]. These findings illustrate a departure from speech perception, where visual speech cues enhance speech intelligibility in noisy auditory environments [54], as they did not enhance speech fluency under adverse speech production circumstances. This may be because visual feedback is not naturally available during speech production, it is less likely integrated with auditory feedback to aid speech monitoring.

### Other factors that affect compensatory responses

Our findings also revealed that repeated exposure to both DAF and PAF reduces the effect of these manipulations on speech. In the DAF experiment, speech rate significantly increased over time for both the no DAF and 200 ms DAF conditions, likely due to participants repeating the same set of 10 alternating sentences throughout the experiment. Similarly, in the PAF experiment, the magnitude of compensatory vocal responses diminished as the experiment progressed. Because both paradigms involved randomized presentation of altered feedback trials and used a constant amount of alterations across trials (i.e., without incremental increases during a ramp phase [9,26,55]), the decline in behavioral effects toward the end of the sessions is more likely attributed to practice effects rather than adaptation to the stimuli.

Interestingly, in the PAF task, this practice effect was more pronounced for the 200-cent perturbation than for the 100-cent perturbation. The 200-cent perturbation not only led to a greater reduction in peak amplitudes but also elicited fewer opposing responses compared to the 100-cent perturbation. Previous research suggests that compensatory opposing responses primarily occur when auditory feedback is perceived as self-generated. Larger perturbations, such as 200 cents, are more likely to be interpreted as external and not self-generated, reducing the likelihood of compensation [56–58]. It might be the case that, for the 200-cent perturbation, participants perceive the auditory feedback as externally generated and develop a strategy to disregard it through practice.

## Conclusion

Our findings underscore the individual variability in susceptibility to altered auditory feedback and demonstrate that susceptibility to DAF does not necessarily predict susceptibility to PAF, supporting the idea that these different auditory feedback manipulations engage distinct compensatory processes and neural mechanisms. Additionally, we tested whether immediate visual feedback through viewing one’s own mouth movements could mitigate the disruptive effects of DAF. In contrary to our expectations, visual feedback did not improve speech fluency but instead exacerbated the effects of DAF, further slowing speech rate. This finding highlights a difference between speech perception and production—while seeing mouth movements aid speech comprehension in noisy environments, they do not necessarily facilitate fluent speech production under adverse auditory feedback conditions.

Together, these findings contribute to our understanding of how individuals process and compensate for auditory feedback disruptions, offering insights into the mechanisms of speech monitoring and control. In future studies, it will be crucial to investigate the neural mechanisms underlying the processing of different auditory feedback manipulations that affect different aspects of speech production—vocalization, articulation and speech coordination. Understanding the structural and functional distinctions among different susceptibility profiles will be crucial for developing strategies that could support speech production in both typical and clinical populations.

## References

[1] Lane H, Webster JW. Speech deterioration in postlingually deafened adults. J Acoust Soc Am. 1991;89(2):859–66. doi: 10.1121/1.1894647 2016436

[2] Waldstein RS. Effects of postlingual deafness on speech production: implications for the role of auditory feedback. J Acoust Soc Am. 1990;88(5):2099–114. doi: 10.1121/1.400107 2269726

[3] Black JW. The effect of delayed side-tone upon vocal rate and intensity. J Speech Disord. 1951;16(1):56–60. doi: 10.1044/jshd.1601.56 14804702

[4] FAIRBANKS G. Selective vocal effects of delayed auditory feedback. J Speech Hear Disord. 1955;20(4):333–46. doi: 10.1044/jshd.2004.333 13272227

[5] Lee BS. Effects of delayed speech feedback. J Acoust Soc Am. 1950;22(6):824–6. doi: 10.1121/1.1906696

[6] Jones JA, Munhall KG. Perceptual calibration of F0 production: evidence from feedback perturbation. J Acoust Soc Am. 2000;108(3 Pt 1):1246–51. doi: 10.1121/1.1288414 11008824

[7] Behroozmand R, Karvelis L, Liu H, Larson CR. Vocalization-induced enhancement of the auditory cortex responsiveness during voice F0 feedback perturbation. Clin Neurophysiol. 2009;120(7):1303–12. doi: 10.1016/j.clinph.2009.04.022 19520602 PMC2710429

[8] Behroozmand R, Korzyukov O, Sattler L, Larson CR. Opposing and following vocal responses to pitch-shifted auditory feedback: evidence for different mechanisms of voice pitch control. J Acoust Soc Am. 2012;132(4):2468–77. doi: 10.1121/1.4746984 23039441 PMC3477187

[9] Behroozmand R, Sangtian S. Neural bases of sensorimotor adaptation in the vocal motor system. Exp Brain Res. 2018;236(7):1881–95. doi: 10.1007/s00221-018-5272-9 29696312

[10] Chen SH, Liu H, Xu Y, Larson CR. Voice F0 responses to pitch-shifted voice feedback during English speech. J Acoust Soc Am. 2007;121(2):1157–63. doi: 10.1121/1.2404624 17348536

[11] Jones JA, Keough D. Auditory-motor mapping for pitch control in singers and nonsingers. Exp Brain Res. 2008;190(3):279–87. doi: 10.1007/s00221-008-1473-y 18592224 PMC2644332

[12] MacDonald E, Munhall KG, editors. A preliminary study of individual responses to real-time pitch and formant perturbations. The Listening Talker: An interdisciplinary workshop on natural and synthetic modification of speech in response to listening conditions; 2012.

[13] Xu Y, Larson CR, Bauer JJ, Hain TC. Compensation for pitch-shifted auditory feedback during the production of Mandarin tone sequences. J Acoust Soc Am. 2004;116(2):1168–78. doi: 10.1121/1.1763952 15376682 PMC1224717

[14] Donath TM, Natke U, Kalveram KT. Effects of frequency-shifted auditory feedback on voice F0 contours in syllables. J Acoust Soc Am. 2002;111(1 Pt 1):357–66. doi: 10.1121/1.1424870 11831808

[15] Burnett TA, Larson CR. Early pitch-shift response is active in both steady and dynamic voice pitch control. J Acoust Soc Am. 2002;112(3 Pt 1):1058–63. doi: 10.1121/1.1487844 12243154

[16] Goldberg TE, Gold JM, Coppola R, Weinberger DR. Unnatural practices, unspeakable actions: a study of delayed auditory feedback in schizophrenia. Am J Psychiatry. 1997;154(6):858–60. doi: 10.1176/ajp.154.6.858 9167517

[17] Lin I-F, Mochida T, Asada K, Ayaya S, Kumagaya S-I, Kato M. Atypical delayed auditory feedback effect and Lombard effect on speech production in high-functioning adults with autism spectrum disorder. Front Hum Neurosci. 2015;9:510. doi: 10.3389/fnhum.2015.00510 26441607 PMC4585204

[18] Boller F, Marcie P. Possible role of abnormal auditory feedback in conduction aphasia. Neuropsychologia. 1978;16(4):521–4. doi: 10.1016/0028-3932(78)90078-7 692865

[19] Kalinowski J, Stuart A. Stuttering amelioration at various auditory feedback delays and speech rates. Eur J Disord Commun. 1996;31(3):259–69. doi: 10.3109/13682829609033157 8944848

[20] Behroozmand R, Phillip L, Johari K, Bonilha L, Rorden C, Hickok G, et al. Sensorimotor impairment of speech auditory feedback processing in aphasia. Neuroimage. 2018;165:102–11. doi: 10.1016/j.neuroimage.2017.10.014 29024793 PMC5732035

[21] Chen X, Zhu X, Wang EQ, Chen L, Li W, Chen Z, et al. Sensorimotor control of vocal pitch production in Parkinson’s disease. Brain Res. 2013;1527:99–107. doi: 10.1016/j.brainres.2013.06.030 23820424

[22] Agnew ZK, McGettigan C, Banks B, Scott SK. Group and individual variability in speech production networks during delayed auditory feedback. J Acoust Soc Am. 2018;143(5):3009. doi: 10.1121/1.5026500 29857719 PMC5963950

[23] Burke BD. Susceptibility to delayed auditory feedback and dependence on auditory or oral sensory feedback. J Commun Disord. 1975;8(1):75–96. doi: 10.1016/0021-9924(75)90028-3 1159107

[24] Howell P, Archer A. Susceptibility to the effects of delayed auditory feedback. Percept Psychophys. 1984;36(3):296–302. doi: 10.3758/bf03206371 6522222

[25] YATES AJ. Delayed auditory feedback. Psychol Bull. 1963;60:213–32. doi: 10.1037/h0044155 14002534

[26] Alemi R, Lehmann A, Deroche MLD. Adaptation to pitch-altered feedback is independent of one’s own voice pitch sensitivity. Sci Rep. 2020;10(1):16860. doi: 10.1038/s41598-020-73932-1 33033324 PMC7544828

[27] Burnett TA, Freedland MB, Larson CR, Hain TC. Voice F0 responses to manipulations in pitch feedback. J Acoust Soc Am. 1998;103(6):3153–61. doi: 10.1121/1.423073 9637026

[28] Burnett TA, Senner JE, Larson CR. Voice F0 responses to pitch-shifted auditory feedback: a preliminary study. J Voice. 1997;11(2):202–11. doi: 10.1016/s0892-1997(97)80079-3 9181544

[29] Lametti DR, Nasir SM, Ostry DJ. Sensory preference in speech production revealed by simultaneous alteration of auditory and somatosensory feedback. J Neurosci. 2012;32(27):9351–8. doi: 10.1523/JNEUROSCI.0404-12.2012 22764242 PMC3404292

[30] Fridriksson J, Hubbard HI, Hudspeth SG, Holland AL, Bonilha L, Fromm D, et al. Speech entrainment enables patients with Broca’s aphasia to produce fluent speech. Brain. 2012;135(Pt 12):3815–29. doi: 10.1093/brain/aws301 23250889 PMC3525061

[31] Snyder GJ, Hough MS, Blanchet P, Ivy LJ, Waddell D. The effects of self-generated synchronous and asynchronous visual speech feedback on overt stuttering frequency. J Commun Disord. 2009;42(3):235–44. doi: 10.1016/j.jcomdis.2009.02.002 19304293

[32] Jones JA, Striemer D. Speech disruption during delayed auditory feedback with simultaneous visual feedback. J Acoust Soc Am. 2007;122(4):EL135-41. doi: 10.1121/1.2772402 17902742 PMC2637445

[33] Ning L-H, Loucks TM, Shih C. Suppression of vocal responses to auditory perturbation with real-time visual feedback. J Acoust Soc Am. 2018;143(6):3698. doi: 10.1121/1.5043383 29960493

[34] Kuznetsova A, Brockhoff PB, Christensen RHB. lmerTest Package: Tests in linear mixed effects models. J Stat Soft. 2017;82(13). doi: 10.18637/jss.v082.i13

[35] Corey DM, Cuddapah VA. Delayed auditory feedback effects during reading and conversation tasks: gender differences in fluent adults. J Fluency Disord. 2008;33(4):291–305. doi: 10.1016/j.jfludis.2008.12.001 19328981

[36] Stuart A, Kalinowski J. Effect of delayed auditory feedback, speech rate, and sex on speech production. Percept Mot Skills. 2015;120(3):747–65. doi: 10.2466/23.25.PMS.120v17x2 26029968

[37] Boersma P. Praat: doing phonetics by computer [Computer program]. http://www praat org/. 2011.

[38] Franken MK, Acheson DJ, McQueen JM, Hagoort P, Eisner F. Opposing and following responses in sensorimotor speech control: Why responses go both ways. Psychon Bull Rev. 2018;25(4):1458–67. doi: 10.3758/s13423-018-1494-x 29869027

[39] Hain TC, Burnett TA, Kiran S, Larson CR, Singh S, Kenney MK. Instructing subjects to make a voluntary response reveals the presence of two components to the audio-vocal reflex. Exp Brain Res. 2000;130(2):133–41. doi: 10.1007/s002219900237 10672466

[40] Guenther FH, Ghosh SS, Tourville JA. Neural modeling and imaging of the cortical interactions underlying syllable production. Brain Lang. 2006;96(3):280–301. doi: 10.1016/j.bandl.2005.06.001 16040108 PMC1473986

[41] Hickok G. The architecture of speech production and the role of the phoneme in speech processing. Lang Cogn Process. 2014;29(1):2–20. doi: 10.1080/01690965.2013.834370 24489420 PMC3904400

[42] Houde JF, Nagarajan SS. Speech production as state feedback control. Front Hum Neurosci. 2011;5:82. doi: 10.3389/fnhum.2011.00082 22046152 PMC3200525

[43] Tourville JA, Guenther FH. The DIVA model: A neural theory of speech acquisition and production. Lang Cogn Process. 2011;26(7):952–81. doi: 10.1080/01690960903498424 23667281 PMC3650855

[44] Meekings S, Scott SK. Error in the superior temporal gyrus? A systematic review and activation likelihood estimation meta-analysis of speech production studies. J Cogn Neurosci. 2021;33(3):422–44. doi: 10.1162/jocn_a_01661 33326327

[45] Floegel M, Fuchs S, Kell CA. Differential contributions of the two cerebral hemispheres to temporal and spectral speech feedback control. Nat Commun. 2020;11(1):2839. doi: 10.1038/s41467-020-16743-2 32503986 PMC7275068

[46] Karlin R, Parrell B. Speakers monitor auditory feedback for temporal alignment and linguistically relevant duration. J Acoust Soc Am. 2022;152(6):3142. doi: 10.1121/10.0015247 36586849 PMC9719414

[47] Oschkinat M, Hoole P. Compensation to real-time temporal auditory feedback perturbation depends on syllable position. J Acoust Soc Am. 2020;148(3):1478. doi: 10.1121/10.0001765 33003874

[48] Oschkinat M, Hoole P. Reactive feedback control and adaptation to perturbed speech timing in stressed and unstressed syllables. J Phon. 2022;91:101133. doi: 10.1016/j.wocn.2022.101133

[49] Oschkinat M, Hoole P, Falk S, Dalla Bella S. Temporal malleability to auditory feedback perturbation is modulated by rhythmic abilities and auditory acuity. Front Hum Neurosci. 2022;16:885074. doi: 10.3389/fnhum.2022.885074 36188179 PMC9519855

[50] Yates AJ. Delayed Auditory Feedback and Shadowing. Quarterly Journal of Experimental Psychology. 1965;17(2):125–31. doi: 10.1080/17470216508416421 14285601

[51] Stuart A, Kalinowski J, Rastatter MP, Lynch K. Effect of delayed auditory feedback on normal speakers at two speech rates. J Acoust Soc Am. 2002;111(5 Pt 1):2237–41. doi: 10.1121/1.1466868 12051443

[52] Takaso H, Eisner F, Wise RJ, Scott SK. The effect of delayed auditory feedback on activity in the temporal lobe while speaking: a positron emission tomography study. 2010. 10.1044/1092-4388(2009/09-0009) PMC4083252 19948756

[53] Chesters J, Baghai-Ravary L, Möttönen R. The effects of delayed auditory and visual feedback on speech production. J Acoust Soc Am. 2015;137(2):873–83. doi: 10.1121/1.4906266 25698020 PMC4477042

[54] Sumby WH, Pollack I. Visual Contribution to Speech Intelligibility in Noise. J Acoust Soc Am. 1954;26(2):212–5. doi: 10.1121/1.1907309

[55] Jones JA, Munhall KG. Remapping auditory-motor representations in voice production. Curr Biol. 2005;15(19):1768–72. doi: 10.1016/j.cub.2005.08.063 16213825

[56] Burnett TA, Freedland MB, Larson CR, Hain TC. Voice F0 responses to manipulations in pitch feedback. J Acoust Soc Am. 1998;103(6):3153–61. doi: 10.1121/1.423073 9637026

[57] Korzyukov O, Bronder A, Lee Y, Patel S, Larson CR. Bioelectrical brain effects of one’s own voice identification in pitch of voice auditory feedback. Neuropsychologia. 2017;101:106–14. doi: 10.1016/j.neuropsychologia.2017.04.035 28461225 PMC5542569

[58] Scheerer NE, Behich J, Liu H, Jones JA. ERP correlates of the magnitude of pitch errors detected in the human voice. Neuroscience. 2013;240:176–85. doi: 10.1016/j.neuroscience.2013.02.054 23466810

